# Expression and clinical significance of CXCR5/CXCL13 in human non-small cell lung carcinoma

**DOI:** 10.3892/ijo.2014.2688

**Published:** 2014-09-30

**Authors:** RAJESH SINGH, PRANAV GUPTA, GOETZ H. KLOECKER, SHAILESH SINGH, JAMES W. LILLARD

**Affiliations:** 1 Department of Microbiology, Biochemistry and Immunology, Morehouse School of Medicine, Atlanta, GA 30310-1495, USA; 2 James Graham Brown Cancer Center, University of Louisville, KY 40202, USA

**Keywords:** chemokine, chemokine receptors, CXCR5, lung cancer, non-small cell lung carcinoma

## Abstract

CXCR5 and/or CXCL13 expression is elevated in certain carcinomas and lymphomas. To determine if these factors are involved in progression of non-small cell lung cancer (LuCa), we evaluated their expression in patients with various forms of this disease. Lung biopsies from patients with non-neoplastic cells (n=8), squamous cell carcinoma (SCC; n=24), or adenocarcinoma (AC; n=54) were stained for CXCR5. Histopathological analysis of these samples showed significantly higher expression of CXCR5 (p<0.001) in carcinomas (i.e., SCCs and ACs) relative to non-neoplastic lung tissue. Nuclear and membrane CXCR5 intensities were highest in ACs, with median values of 185 and 130, respectively, followed by SCCs with median values of 170 and 110, respectively. The lowest nuclear and membrane expressions of CXCR5 were found in non-neoplastic tissues, having median values of 142 and 90, respectively. Sera from SCC patients (n=17), AC patients (n=14), and healthy controls (n=9) were tested for the presence of CXCL13. Serum CXCL13 levels in LuCa patients were higher than in healthy controls. CXCR5 expression in cell lines of human non-small cell lung carcinoma (NCI-H1915) and small cell lung carcinoma (SW-1271) were evaluated by flow cytometry. CXCR5 expression was higher in NCI-H1915 cells relative to SW-1271 cells. The functional significance of CXCR5 expression was tested in a migration assay. In response to CXCL13, more NCI-H1915 cells migrated than SW-1271 cells. These findings suggest that the CXCR5-CXCL13 axis influences LuCa progression. After validation in larger patient groups, CXCR5 and CXCL13 may prove useful as biomarkers for LuCa. Correspondingly, blockade of this axis could serve as an effective therapy for LuCa.

## Introduction

Lung cancer (LuCa) is the leading cause of cancer-related deaths among men and women worldwide and is responsible for more deaths than breast, colon, and prostate cancer (PCa) combined (1). LuCas are broadly classified into small cell lung carcinomas (SCLCs) and non-small cell lung carcinomas (NSCLCs). About 85% of all LuCas are identified as NSCLCs; 75% of these are metastatic or advanced at diagnosis (2). Although patients presenting with stage I–II diseases are usually treated with surgery, half of these cases subsequently develop metastatic disease that proves to be fatal. Despite many efforts, little has been achieved for the treatment of this deadly disease. Advances in understanding the factors involved in LuCa progression and development of prognostic and predictive markers have the potential to improve therapeutic outcomes.

NSCLC growth and metastases to secondary sites are highly regulated events, which involve cellular transformation, establishment of a pro-angiogenic environment, and migration and invasion of tumor cells. This latter process is analogous to leukocyte trafficking. To this end, chemokines and their receptors play a major role. Chemokines are small, 8–10 kDa proteins involved in directional migration of cells towards a chemokine gradient that is detected by G-protein-coupled chemokine receptors. These chemotactic cytokines are further classified into CC, CXC, C and CX3C family members, based on their cysteine residues and disulfide bonds. These chemokines are essential for homeostasis and function of the immune and stem cell systems. In recent years, a new role of chemokines has emerged, which involves neoplastic transformation of cells, tumor cell growth and survival, and organ-specific metastasis during carcinogenesis (3,4). Of the CXC chemokines, CXCR4 is involved in NSCLC progression. NSCLC tumors and cell lines express CXCR4, and, in mouse models, anti-CXCR4 antibody reduces tumor metastases. Further, organs to which NSCLCs preferentially metastasize constitutively express CXCL12, a natural ligand for CXCR4 (5). To our knowledge, this is the first study to demonstrate the association of CXCR5 and CXCL13 with NSCLC. In contrast to CXCR4, which is expressed by normal and malignant hematopoietic and non-hematopoietic cells (6), CXCR5 is expressed primarily by mature, recirculating B cells and by small subsets of CD4^+^ and CD8^+^ T cells. The migration of these leukocytes into and within lymph nodes is controlled by CXCR5-CXCL13 interactions (6,7). Recently, it has been recognized that the CXCR5-CXCL13 axis is associated with various hematologic (7–10) and solid tumor malignancies (11–16). Indeed, CXCR5 and CXCL13 are expressed in prostate, breast, neuronal, and oral carcinomas (11,13–15). Previously, we elucidated the molecular mechanisms and functional significance of CXCR5 and CXCL13, whereby this axis promotes PCa cell migration, invasion, and differential matrix metalloproteinase (MMP) expression (17). We also showed that CXCL13-mediated invasion of PCa cells requires Akt and ERK1/2 activation, suggesting a new role for DOCK2, a protein involved in intracellular signaling, in proliferation of hormone-refractory CXCR5-positive PCa cells (18).

Based on these findings, we investigated the expression of CXCR5 and CXCL13 in patient samples of NSCLCs, evaluating the expression of CXCR5 in normal, squamous cell carcinoma (SCC), and adenocarcinoma (AC) tissues by immunohistochemical staining. To determine the association of CXCL13 with NSCLC progression, serum CXCL13 levels were analyzed for both subtypes of NSCLCs. Furthermore, the expression patterns of CXCR5 in human LuCa cell lines were determined, and the findings were correlated with clinicopathological features to evaluate the role of CXCR5 in NSCLC progression. To understand the biological significance of CXCR5 over-expression in NSCLCs, the migration potential of LuCa cells via CXCL13 was analyzed. These results demonstrate the association with and point to a role of CXCR5 and CXCL13 in NSCLCs.

## Materials and methods

### Tissue specimens

Tissue microarray (TMA) slides containing malignant (n=78), non-neoplastic (n=8), and other (n=12) lung tissues (n=98) were purchased from AccuMax Array (ISU Abxis Co., Ltd., Seoul, Korea). These spots were generated from lung biopsies of 45 cases diagnosed with NSCLCs with histological subtypes of AC (n=27), SCC (n=12), and others (n=6); and eight non-neoplastic cases. To construct TMAs, two cores (1 mm in diameter) per patient were arrayed on a receiver paraffin block, and, concerning the histopathology, a qualified pathologist validated each core of the TMAs twice for class and grade of the tumor. LuCa TMAs consisted of tumors from 45 patients and represented all histopathological subtypes reported for LuCa. The total of 98 spots represented eight non-neoplastic, 54 ACs, 24 SCCs, and 12 others.

### Immunohistochemistry

TMA slides containing biopsies obtained from malignant and non-neoplastic cases were stained for CXCR5. Briefly, TMAs were de-paraffinized in xylene and rehydrated through a graded series of ethanol (100, 95 and 70%) for 5 min in each series and washed in distilled water. After de-paraffinization, antigen retrieval was accomplished by incubating TMAs with 0.01 M EDTA (pH 8.0) in a pressure cooker for 5 min. Slides were then cooled in running water and transferred to Tris-buffer (pH 7.6). The endogenous peroxidase activity was blocked by incubating the slides with 3% H_2_O_2_ in phosphate-buffered saline (PBS) for 5 min. The slides were then rinsed three times with de-ionized water followed by Tris-buffer (pH 7.6). Following washing, Fc blocking was accomplished by incubating the slides with Fc Block (Innovex Biosciences, Inc., Richmond, CA, USA) for 30 min at room temperature (RT) in a humidity chamber. To reduce non-specific binding, the sections were washed with Tris-buffer and incubated with 3% normal goat serum for 1 h at RT. The slides were then washed with Tris-buffer, and sections were incubated with 5 μg/ml of HRP-conjugated mouse anti-CXCR5 antibody (R&D Systems, Minneapolis, MN, USA) for 90 min at RT in a humidity chamber. Negative control slides were incubated with 5 μg/ml of mouse isotype control antibody (R&D Systems). Following incubation, sections were washed with Tris-buffer and developed with a 3,3′-diaminobenzidine (DAB) (Vector Laboratories, Inc., Burlingame, CA, USA) as a chromogen for 25 min at RT. The sections were also incubated with alkaline phosphatase (AP)-conjugated goat anti-mouse antibody (Invitrogen Life Technologies, Grand Island, NY, USA) for 20 min at RT and developed with AP-New Magenta (BioFX Laboratories, Inc., Owings Mills, MD, USA) substrate for 25 min at RT. Counterstaining was with hematoxylin (Sigma, St. Louis, MO, USA). Subsequently, sections were washed with water, dehydrated in 70, 95%, and absolute alcohol for 5 min each, passed through xylene three times for 1 min each, and mounted with Permount (Sigma). The immunopositivity of the sections was analyzed using an Aperio ScanScope scanning system (Aperio Technologies, Vista, CA, USA).

### Quantitation of immunohistochemical staining

To analyze the immunohistochemical staining, virtual slides were created from the stained samples after scanning each specimen with an Aperio ScanScope scanning system (Aperio Technologies). The ScanScope generated true-color digital images of each stained sample, which were viewed using Aperio ImageScope v.6.25 software. The algorithm for determining the intensity of membrane-specific staining was used to calculate, for each sample, the staining intensity and percent of target labeled by digitally analyzing the color intensity. A color markup image for each slide was obtained based on membrane staining intensity. The output was viewed as determinations of staining intensity ranging from 0–3 to correlate with conventional manual scoring methods (where 0, negative and 3, strong staining), and statistical analyses were performed using the means of these values.

### Quantitative enzyme-linked immunosorbent assay (ELISA) for serum CXCL13

Sera from patients with SCCs (n=17), ACs (n=14), and healthy controls (n=9) were obtained from the James Graham Brown Cancer Center, University of Louisville, KY, USA. Healthy donors had no active lung disease or symptoms at the time of blood collection. All subjects gave written informed consent and were approved by the University of Alabama at Birmingham Institutional Review Board (IRB). Subsequently, the University of Louisville IRB approved the use of these diagnostic specimens in accordance with the Department of Health and Human Service Policy for the Protection of Human Research Subjects 45 CFR 46.101(b) 2 and use of archived de-identified materials. Serum CXCL13 levels were measured by human CXCL13 Quantikine ELISA (R&D Systems), following the manufacturer’s instructions. Briefly, 100 μl of assay diluent (provided with the kit), followed by 50 μl of standard, control, or samples (sera from patients and healthy controls) were added in different wells of a 96-well plate and incubated for 2 h at RT. Following washing four times with Quantikine Wash Buffer 1 (provided with the kit), 200 μl of conjugate (antibody) was added to each well, and the plate was incubated for 2 h at RT. The plate was washed further, and 200 μl of substrate solution (provided with the kit) was added to each well. The plate was incubated for 30 min in the dark at RT. Following incubation, 50 μl of stop solution was added to each well, and the plate was read in an ELISA reader at 450 nm. The ELISA assays were capable of detecting >1 pg/ml of CXCL13.

### Cell cultures

Human NSCLC NCI-H1915 (CRL-5904) and small cell lung carcinoma SW-1271 (CRL-2177) cell lines were obtained from American Type Cell Culture (ATCC) (Teddington, UK). NCI-H1915 cells were cultured in RPMI-1640 media (Mediatech Cellgro, Herndon, VA, USA) supplemented with 10% heat-inactivated fetal bovine serum (FBS) (Sigma), 100 μg/ml streptomycin, and 100 U/ml penicillin (Sigma) at 37°C with 5% CO_2_. SW-1271 cells were cultured in Leibovitz’s L-15 Medium (Mediatech Cellgro) supplemented with 10% FBS, 100 μg/ml streptomycin, and 100 U/ml penicillin at 37°C with 100% air. Cells were split twice a week to ensure optimal growth conditions.

### Functional analysis of CXCR5 expression

For both LuCa cell lines, CXCR5 surface expression was analyzed by flow cytometry. Briefly, LuCa cells were washed three times in PBS [supplemented with 1% bovine serum albumin (BSA)], and treated with 1 μg of Fc Block (BD Biosciences, San Jose, CA, USA) per 10^5^ cells for 15 min at RT. Fc-blocked cells were incubated with 1 μg of fluorescein-conjugated mouse anti-human CXCR5 or fluorescein-conjugated mouse IgG2a isotype control antibodies (R&D Systems) per 10^5^ cells for 1 h at 4°C. Following staining, the unbound antibodies were removed by washing the cells three times with fluorescence-activated cell-sorting (FACS) buffer (1% BSA in PBS). The labeled cells were then fixed in 500 μl of 2% paraformaldehyde solution and analyzed by flow cytometry using a FACScan flow cytometer (BD Biosciences). The flow cytometry data were analyzed with FlowJo software. The stained cells were also analyzed by an Amnis ImageStream system (Amnis Corp., Seattle, WA, USA).

### Migration assay

Cell migration was assessed with a BD BioCoat™ migration chamber system (BD Biosciences). Briefly, Matrigel inserts were hydrated for 2 h with 500 μl of serum-free Dulbecco’s modified Eagle’s medium at 37°C with 5% CO_2_. CXCL13 (PeproTech, Rocky Hill, NJ, USA), at a concentration of 0 or 100 ng/ml, was added to the bottom chamber containing serum-free RPMI medium. Next, NCI-H1915 and SW-1271 cells were incubated with isotype control or anti-human CXCR5 antibody at a concentration of 1 μg/ml (both from R&D Systems) for 1 h at 37°C with 5% CO_2_ and added to the top chambers in serum-free RPMI medium at 10,000 cells per well. The cells were allowed to migrate for 8 h at 37°C under 5% CO_2_. Non-migrating cells on the upper surface of the membrane were removed with a cotton swab. The cells that migrated to the lower surface were fixed with 100% methanol for 5 min at RT, stained with crystal violet for 2 min, and rinsed twice with distilled water. The membranes were placed on glass slides. The migrated cells were counted by microscopy at ×40 magnification. The experiments were performed in triplicate and repeated three times.

### Statistics

The intensity of CXCR5 and CXCL13 expression by lung TMAs was tested for normality assumptions using the Shapiro-Wilk test and was transformed to a Log scale. The general linear models procedure was used to test the association of CXCR5 and CXCL13 expression and disease condition using SAS v.9.1.3 statistical analysis software. Results were declared significant at α level of 0.001. The experimental data were compared using a two-tailed Student’s t-test and expressed as mean ± SEM. The results were analyzed using the Stat View program (Abacus Concepts, Inc., Piscataway, NJ, USA) and were labeled statistically significant if p-values were <0.01. With Cell Quest Software, the Kolmogorov-Smirnov (K-S) two-sample test was used to calculate the statistical significance of the CXCR5 histograms.

## Results

### Immunohistochemical analyses demonstrate CXCR5 is overexpressed in LuCa tissues relative to non-neoplastic tissues

**Figure 1:**
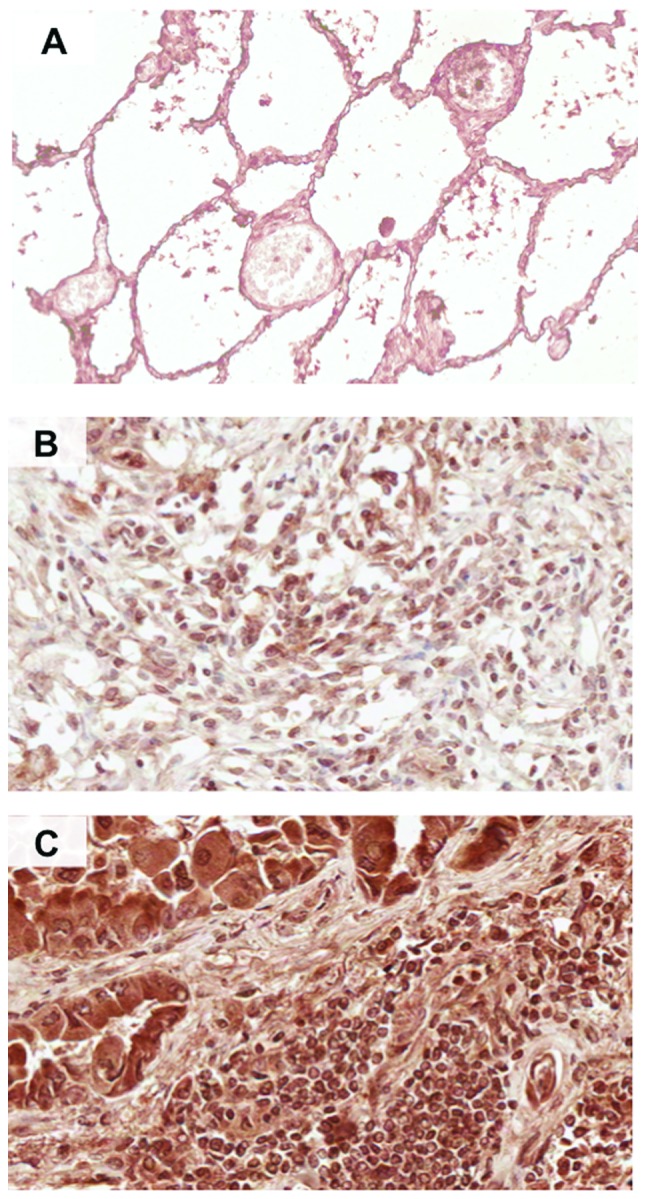
Immunohistochemical detection of CXCR5 in lung cancer (LuCa) tissues. Representative figures of (A) non-neoplastic (n=8), (B) squamous cell carcinoma (SCC) (n=24) and (C) adenocarcinoma (AC) (n=54) lung tissues stained with anti-CXCR5 antibodies. Brown [3,3′-diaminobenzidine (DAB)] color shows CXCR5 staining. The images were captured using an Aperio ScanScope CS system with a 40× objective. Immuno-intensities of CXCR5 in each section were quantified using image analysis Aperio ImageScope v.6.25 software.

**Figure 2:**
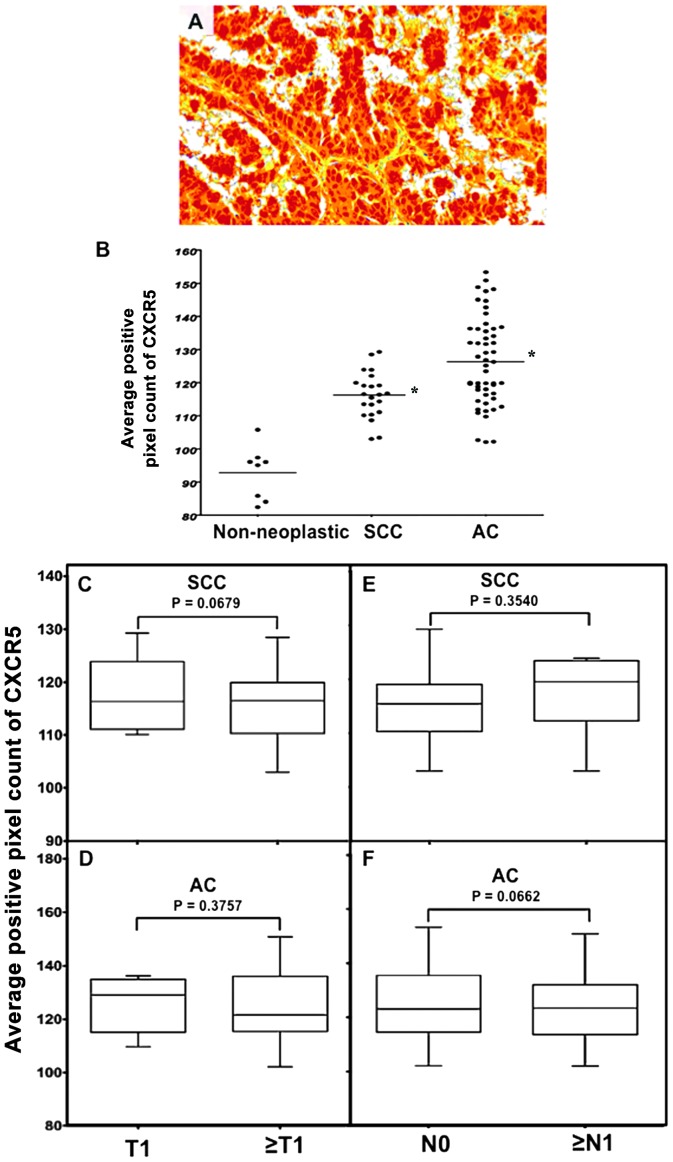
CXCR5 expression by lung cancer (LuCa) tissues. (A) Representative figures of non-neoplastic (n=8), squamous cell carcinoma (SCC) (n=22), and adenocarcinoma (AC) (n=52) lung tissues stained with isotype control or anti-CXCR5 antibodies. Positive pixel counts were quantified using a membrane algorithm of image analysis Aperio ImageScope v.6.25 software. (B) The average positive pixel count of CXCR5, in non-neoplastic (n=8), SCC (n=22), and AC (n=52) tissues, quantified with a nuclear algorithm of image analysis Aperio ImageScope v.6.25 software. ^*^Significant differences (p<0.001) between groups with LuCa and control. (C–F) Average pixel counts for CXCR5 for different tumor stages and for tumors with nodal involvement in SCC and AC cases, respectively.

**Figure 3:**
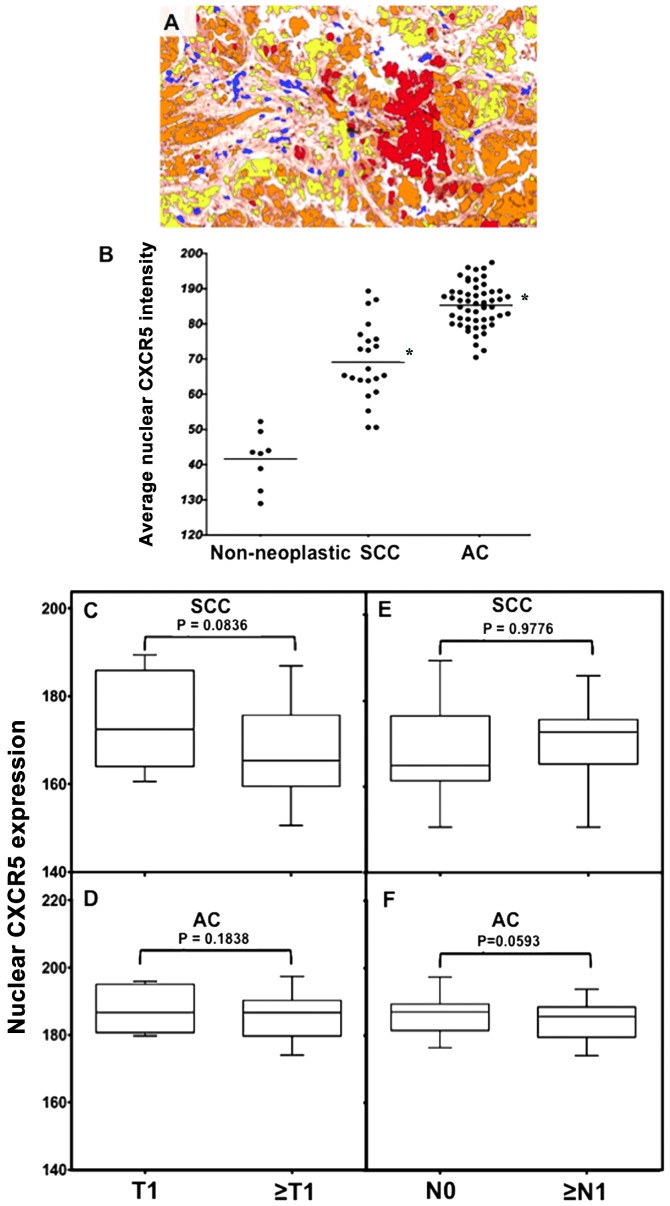
Nuclear CXCR5 expression in lung cancer (LuCa) tissues. (A) Representative figures of non-neoplastic (n=8), squamous cell carcinoma (SCC) (n=22), and adenocarcinoma (AC) (n=52) lung tissues stained with isotype control or anti-CXCR5 antibodies. Brown [3,3′-diaminobenzidine (DAB)] color show CXCR5 staining. An Aperio ScanScope CS system with a 40× objective captured digital images of each slide. Stained cells with negative and positive nuclei were counted and categorized according to stain intensity 0 (Blue), 1+(yellow), 2+(orange) and 3+(Red). (B) The nuclear intensity of CXCR5, in non-neoplastic (n=8), SCC (n=22), and AC (n=52) tissues, quantified using a nuclear algorithm of image analysis Aperio ImageScope v.6.25 software. ^*^Significant differences (p<0.001) between groups with LuCa and control. (C–F) Nuclear intensities of CXCR5 in different tumor stages and in tumors with nodal involvement in SCC and AC cases, respectively.

**Figure 4:**
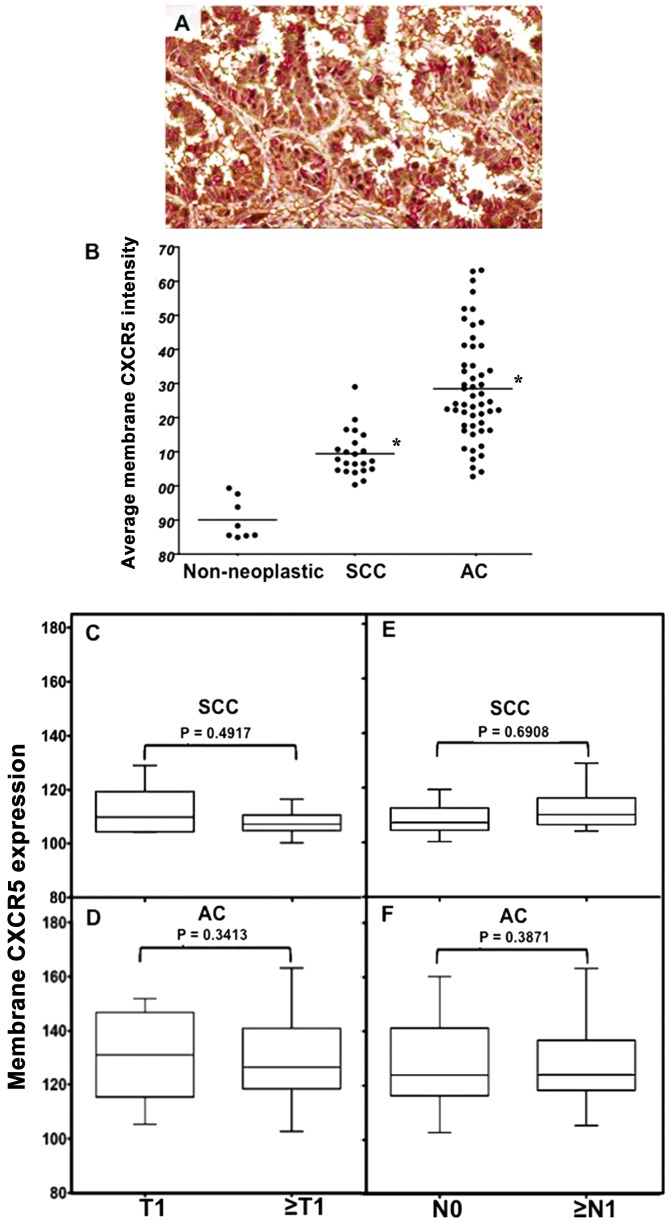
Membrane CXCR5 expression by lung cancer (LuCa) tissues. (A) Representative figure of non-neoplastic (n=8), squamous cell carcinoma (SCC) (n=22) and adenocarcinoma (AC) (n=52) lung tissues stained with isotype control or anti-CXCR5 antibodies. Brown [3,3′-diaminobenzidine (DAB)] color show CXCR5 staining. An Aperio ScanScope CS system with a 40× objective captured digital images of each slide. The membrane intensity of CXCR5 was quantified using a membrane algorithm of image analysis Aperio ImageScope v.6.25 software. (B) The membrane intensity of CXCR5, in non-neoplastic (n=8), SCC (n=22), and AC (n=52) tissues, quantified using a nuclear algorithm of image analysis Aperio ImageScope v.6.25 software. ^*^Significant differences (p<0.001) between groups with LuCa and control. (C–F) Membrane intensities of CXCR5 for different tumor stages and for tumors with nodal involvement in SCC and AC cases, respectively.

LuCa TMAs, consisting of 98 biopsy areas, generated from biopsies of malignant (SCC and AC) and non-neoplastic cases, were analyzed for CXCR5 expression by immunohistochemistry. CXCR5 was expressed in tissues collected from SCC and AC cases (p<0.001) relative to non-neoplastic tissues, in which no signal was detected (Fig. 1). Average positive, nuclear, and membrane CXCR5 intensities were quantified in non-neoplastic, SCC, and AC cases using image analysis Aperio ImageScope v.6.25 software (Figs. 2–4). These intensities were highest in ACs, with median values of 128, 185, and 130, respectively; followed by SCC with median values of 118, 170, and 110, respectively; and lowest in non-neoplastic tissues with median values of 92, 142, and 90, respectively. Further, CXCR5 expression correlated with stage (T) and nodal involvement (N) of tumors in both SCC and AC tissues. In SCCs, total average CXCR5 expression in cases with T1 (median value, 116) was essentially equal to cases with ≥T2 (median value, 115) but lower than ≥T2 in ACs (median value, 120). However, the average positive pixel count of CXCR5 expression in ACs was higher in T1 (average value, 154) than in ≥T2 (average value, 138) there was higher CXCR5 expression for cases with ≥N1 (median value, 121) than N0 (median value, 116) in SCCs, but there was little difference for AC cases (median value 124) (Fig. 2). Both nuclear and membrane CXCR5 expression was higher in T1 than in ≥T2 SCCs (median values, 174 and 110 vs. 162 and 108, respectively). Although nuclear and membrane CXCR5 expressions in T1 (median values, 186 and 132, respectively) were slightly higher than ≥T2 (median values 185 and 128, respectively) of ACs, similar to SCCs, the maximum expressions of both nuclear and membrane CXCR5 intensities in ≥T2 ACs (average values 198 and 162, respectively) were higher relative to the maximum for T1 ACs (average values, 194 and 150, respectively). Further, both nuclear and membrane CXCR5 intensities were higher in SCCs with ≥N1 (median values, 172 and 112) relative to SCCs with N0 (median values, 162 and 108). However, in AC tissues, both nuclear and membrane CXCR5 intensities were essentially equal in N0 and ≥N1, with median values of 186 and 122, respectively (Figs. 3 and 4).

### Levels of serum CXCL13 are elevated in LuCa patients

**Figure 5:**
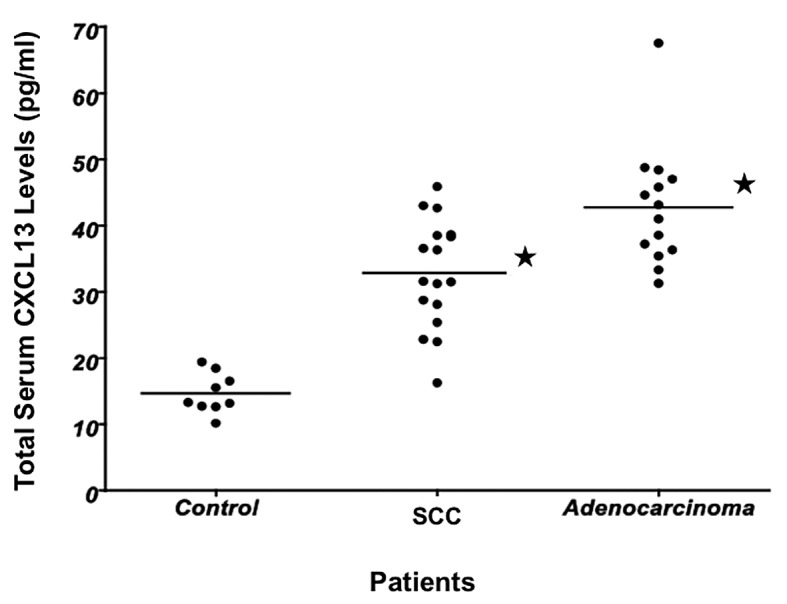
Level of serum CXCL13 in lung cancer (LuCa) patients. Serum CXCL13 levels in normal healthy donors (n=9) and patients diagnosed with squamous cell carcinoma (SCC) (n=17) or adenocarcinoma (AC) (n=14) were analyzed by a quantitative enzyme-linked immunosorbent assay (ELISA) technique, which was capable of detecting >5 pg/ml. Solid circles indicate individual serum CXCL13 levels and lines show median concentrations of each group. ^*^Significant differences (p<0.01) between groups with LuCa and control.

Serum CXCL13 in SCC and AC patients was quantified with a commercially available ELISA kit. Similar to histo-pathological findings of CXCR5, the serum CXCL13 level was significantly higher for AC followed by SCC patients, relative to healthy controls. The median values of CXCL13 expression (pg/ml) in serum of ACs and SCCs were 42 and 32, respectively (Fig. 5). However, the CXCL13 expression in healthy controls was lower, with a median value of 15.

### CXCR5 is over-expressed on LuCa cell lines

**Figure 6:**
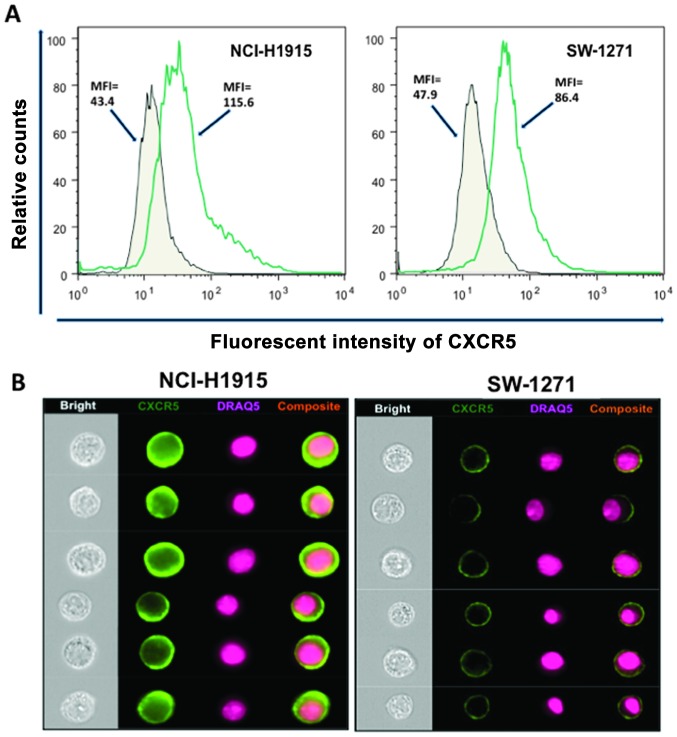
Expression of CXCR5 in lung cancer (LuCa) cells. (A) Non-small cell lung carcinoma (NCI-H1915) and small cell lung carcinoma (SW-1271) cells were stained with FITC-conjugated isotype control antibodies (solid histogram) or FITC-conjugated anti-CXCR5 (open histogram) and quantified in triplicates by flow cytometry. The experiments were repeated three times. (B) NCI-H1915 and SW-1271 cells were stained with FITC-conjugated anti-CXCR5 or FITC-conjugated isotype control antibodies. Images were acquired by multispectral imaging flow cytometry.

CXCR5 expression was evaluated in human NSCLC (NCI-H1915) and SCLC (SW-1271) cell lines using flow cytometry and images were captured by a flow-based imaging system (Amnis ImageStream system). Both cell lines stained positive for CXCR5 and a nuclear stain, DRAQ5. The intensity of membrane CXCR5 expression, measured in terms of mean fluorescence intensity (MFI), was higher in NSCLC (NCI-H1915) cells, relative to SCLC (SW-1271) cells (MFI, 115.6 vs. 86.4) (Fig. 6A). CXCR5 was expressed on membranes of both cell lines, as seen by their composite images (Fig. 6B).

### LuCa cells migrate following the CXCR5-CXCL13 interaction

**Figure 7:**
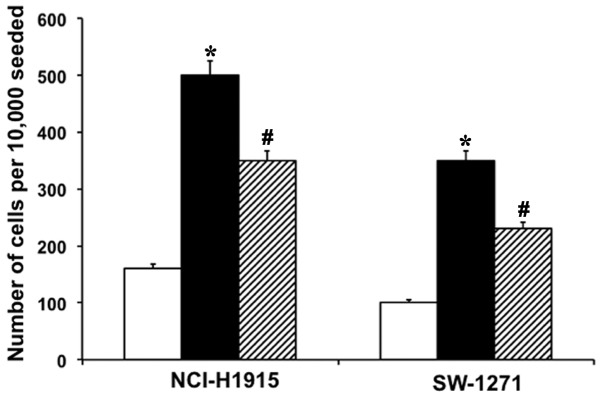
CXCL13 mediated lung cancer (LuCa) cell migration. NSCLC (NCI-H1915) and SCLC (SW-1271) cells were tested for their capacity to migrate toward the chemotactic gradients of 0 (open boxes) or 100 ng/ml of CXCL13 (closed boxes). One group of cells was treated with anti-human CXCR5 antibody (1 μg/ml, hashed boxes) before the migration assay. ^*^Significant differences (p<0.01) between no addition and addition and chemokine-induced cells. ^#^Significant difference between chemokine engagement and receptor neutralization.

The functional significance of CXCR5 expression by LuCa cell lines was demonstrated by the capacity of NCI-H1915 and SW-1271 cells to migrate towards CXCL13. Both types of cells migrated to media with CXCL13, relative to media without CXCL13 (Fig. 7). However, the numbers of NCI-H1915 cells that migrated in response to CXCL13 was higher than the numbers of SW-1271 cells. CXCR5-CXCL13 dependent chemotaxis was abrogated by anti-CXCR5 antibodies. Cells treated with isotype control antibodies served as controls, in which no inhibition in the number of migratory cells was observed in response to CXCL13.

## Discussion

Chemokines/chemokine receptors apparently facilitate tumor dissemination, leading to metastasis, growth, and cell survival (19–21). Few studies have addressed the significance of chemokine/chemokine receptor expression in NSCLCs. Higher expressions of CXCR1, CXCR2, and CXCR4 with their ligands CXCL5, CXCL8, and CXCL12 appear to be associated with tumor angiogenesis, metastasis, and poor survival in NSCLCs (5,22–26). Until now, there were no comprehensive studies regarding the presence or potential role of CXCR5 in NSCLCs.

CXCR5 is a principal regulator for targeting T cells, B cells, and dendritic cells into secondary lymphoid organs. The CXCR5-CXCL13 axis is involved in development and progression of solid tumors, e.g., breast cancers and neuronal cancers (13,14). Recently, we demonstrated a differential expression of CXCR5 in PCa cell lines correlated with PCa progression (17,18). These findings provided the rationale for the present study, which characterizes CXCL5 and CXCR13 expression and interactions during LuCa progression.

The expression of CXCR5 and/or CXCL13 was assessed in LuCa tissues, serum, and LuCa cell lines. Higher CXCR5 expression in LuCa tissues, relative to non-neoplastic tissues and elevated serum CXCL13 in LuCa patients relative to healthy controls correlated with disease progression. SCCs and ACs are two major histologic types of NSCLCs. Patients with ACs have a poorer prognosis than those with SCCs (27,28). However, the differences in biological aggressiveness between SCCs and ACs of the lung are not well understood. We found higher nuclear and membrane intensities of CXCR5 in ACs relative to SCCs. Our findings demonstrate that CXCR5 is differentially expressed in lung carcinomas depending on stage of the disease. Moreover, differential nuclear and membrane CXCR5 expression in SCCs and ACs correlate with their aggressiveness. We further investigated CXCR5 expression in relation to tumor stage (T) and nodal metastasis (N). Although we did not find a statistically significant correlation between CXCR5 expression and tumor stage or nodal metastasis, there was increased CXCR5 expression in SCC cases with nodal metastases. Hence, it is plausible that the CXCR5-CXCL13 axis is involved in tumor dissemination to lymph nodes. We did not observe any distinct change in CXCR5 expression in AC cases with increased tumor stage and nodal metastasis. Since the present study was limited to a small number of patients, subsequent studies with more patient samples from each group (SCC and AC) and subgroups (T1, ≥T2 and N0 and ≥N1) will provide conclusive information correlating CXCR5 expression in NSCLCs with disease progression and survival.

In our previous studies, we showed that serum CXCL13 levels correlated with prostatic disease and mediated PCa cell migration, integrin clustering, and cell adhesion (12). Here, for NSCLC patients, serum CXCL13 levels in ACs were higher than in SCCs, which may be associated with the aggressiveness of the latter disease.

In view of the expression of CXCR5 in LuCa tissues and to understand the biological significance of CXCR5 expression, we stained and analyzed CXCR5 expression on two human LuCa cell lines, NSCLC (NCI-H1915) and SCLC (SW-1271). We also determined the migration potential of these cells under a chemotactic gradient of CXCL13. Staining and migration assay results were in accordance with the hypothesis that the CXCR5-CXCL13 axis is involved in LuCa cell dissemination and/or metastasis. SW-1271 cells, which had lower expression of CXCR5 relative to NCI-H1915 cells, were not as responsive to CXCL13 as were NCI-H1915 cells.

In conclusion, NSCLC tissues expressed CXCR5, which correlated with stage/grade of the disease. Higher CXCR5 expression and migration by NSCLC cells suggest a role in migration and metastasis of primary lung tumors in response to CXCL13. These findings indicate that differential expression patterns of CXCR5 and CXCL13 in two subtypes (SCC and AC) of NSCLC are associated with differences in their prognosis and survival. Identification of a sensitive tumor marker, predictive of diagnosis, prognosis, and drug sensitivity, would enhance NSCLC treatment and diagnosis. We propose that CXCR5/CXCL13, either alone or in combination, could be used as a prognostic biomarker for LuCa; however, this can be established only by larger studies in which these factors are evaluated in the same patients.
